# Hémorragies digestives hautes révélant une sangsue

**DOI:** 10.11604/pamj.2015.22.155.7837

**Published:** 2015-10-16

**Authors:** Djafar Mamoudou, Mahamadou Diarra, Rabiou Ahidan, Moumouni Garba, Mounia Idrissi, Moustapha Hida

**Affiliations:** 1 Service de Pédiatrie, CHU Hassan II, Fès, Maroc; 2 Service de Pédiatrie, HNN, Niamey, Niger

**Keywords:** Hémorragies digestives, hématémèses, sangsue, endoscopie, GI bleeding, haematemesis, leech, endoscopy

## Abstract

Les hémorragies digestives hautes sont un motif fréquent de consultation aux urgences pédiatriques et font généralement suite à des causes multiples comme les œsophagites, gastrites. Cependant son étiologie liée à l'ingestion accidentelle d'un corps étranger comme la sangsue est rarement décrite. Nous rapportons le cas d'un enfant de 3ans admis aux urgences pédiatriques pour des hémorragies digestives hautes chez qui l'endoscopie digestive avait mis en évidence une sangsue enclavée derrière la glotte. La prise en charge consistait en une extraction de ce corps étranger avec surveillance des constantes vitales.

## Introduction

Les sangsues sont des vers marins ou d'eau douce appartenant à la classe des hirudinées et à l'embranchement des annélides. Il en existe près de 127 genres et plus de 300 espèces [1]. La contamination est souvent secondaire à l'ingestion d'une eau provenant d'un étang ou d'un marais. Il a été décrite des cas d'infestation au Maroc, notamment dans la région de Fès [1], dans des pays d'Asie [2–5]. La symptomatologie clinique est dominée par le syndrome hémorragique. Nous rapportons le cas d'un enfant de 3 ans admis aux urgences pédiatriques dans un tableau d'hémorragie digestive haute causé par l'ingestion accidentelle d'une sangsue.

## Patient et observation

**Figure 1:**
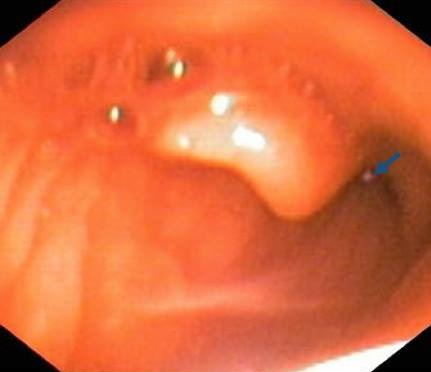
Vue endoscopique montrant la présence d'une sangsue enclavée derrière la luette (flèche)

**Figure 2:**
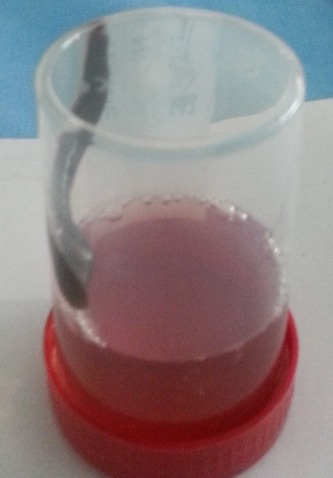
Image de la sangsue après extraction

Un enfant de 3 ans, issue du milieu rural, a été admis aux urgences pédiatriques pour des hématémèses associée à des melæna dans un contexte d’épigastralgie évoluant depuis cinq jours. L'interrogatoire avait révélé une notion d'ingestion d'eau de puits un jour avant l'apparition de la symptomatologie. L'examen clinique à l'admission avait trouvé un enfant en bon état général, eupnéique, stable sur le plan hémodynamique avec des conjonctives et muqueuse moyennement colorées. La numération sanguine objectivait une anémie avec un taux d'hémoglobine à 8,3g/dl. Une exploration endoscopique a été réalisée dans le cadre du bilan étiologique et avait permis de mettre en évidence un corps étranger dont l'extraction a été faite à l'aide d'une pince Magill, sans incidents (Figure 1). Son examen avait montré qu'il s'agissait d'une sangsue de 5cm de longueur et 1,5 cm de largeur (Figure 2). La suite de l'exploration endoscopique avait mis en évidence une muqueuse œso-gastro-duodénale d'aspect normal avec présence de sang noir dans l'estomac. L’évolution après extraction était favorable.

## Discussion

Les hémorragies digestives hautes secondaires à l′ingestion accidentelle de sangsue sont rarement decrites dans la littérature. La localisation la plus fréquente est nasale, et est à l'origine d'obstruction nasale et d’épistaxis récidivantes [2–4], des hématémèses et obstructions des voies respiratoires dans la localisation oro-laryngée, tandis qu'au niveau du tube digestif les hématémèses et/ou melæna dominent le tableau clinique [3, 5, 6]. Il s'agit d'une affection qui touche les sujets qui vivent dans les zones ou l'accès à l'eau potable est difficile comme rapporté dans notre observation. Le syndrome hémorragique constitue le motif de consultation le plus fréquent. Il peut s'agir d'une hémorragie digestive généralement haute, souvent associés à de melæna en fonction de l’évolution du tableau clinique dans le temps comme c'est le cas chez notre patient qui n'a consulté que cinq jours après le début du premier symptôme. Ainsi devant un enfant qui présente une hémorragie digestive haute, l′exploration endoscopique est une étape capitale dans la prise en charge. Elle permettra non seulement de déterminer la cause (corps étranger, ou autres choses) mais aussi de réaliser des gestes thérapeutiques comme l'extraction de la sangsue réalisée chez notre patient. Le geste est réalisé sous anesthésie générale au mieux chez un enfant en position latérale de sécurité pour éviter l'inondation des voies aériennes par du sang provenant de la voie digestive. L'hémostase est généralement obtenu après extraction de la sangsue et le patient sort après une surveillance des paramètres vitaux.

## Conclusion

L'ingestion des sangsues est rarement décrite dans la littérature comme cause d′hémorragies digestives hautes chez l'enfant. L'indication d'une exploration endoscopique ne doit souffrir d'aucun retard. Elle permettra le diagnostic étiologique et la prise en charge. Le geste est réalisable sans incidents dans les mains d'une équipe rompue à cette technique.
